# Dataset for genotyping validation of *cytochrome P450 2A6* whole-gene deletion (CYP2A6*4) by real-time polymerase chain reaction platforms

**DOI:** 10.1016/j.dib.2015.10.019

**Published:** 2015-10-25

**Authors:** Makiko Shimizu, Tomoki Koyama, Izumi Kishimoto, Hiroshi Yamazaki

**Affiliations:** Laboratory of Drug Metabolism and Pharmacokinetics, Showa Pharmaceutical University, Machida, Tokyo 194-8543, Japan

**Keywords:** CYP2A6*1, CYP2A6*4, Copy number assays, Real-time PCR, TaqMan, Whole-gene deletion

## Abstract

This data article contains a supplementary figure and validation data relating to the research article entitled “Genotyping of wild-type *cytochrome P450 2A6* and whole-gene deletion using human blood samples and a multiplex real-time polymerase chain reaction method with dual-labeled probes” (Shimizu et al., Clinica Chimica Acta 441, 71–74, 2015), which presents a multiplex real-time polymerase chain reaction method with dual-labeled probes for human *P450 2A6* wild-type and whole-gene deletion. Real-time methods have dramatically improved the speed of complex genetic diagnostics compared to conventional assays based on restriction enzyme digestion. Here, we show the basic assay validation data by single and multiplex determinations in comparison with commercial TaqMan copy number assays for *P450 2A6*.

**Specifications table**

**Table:** 

Subject area	Biology

More specific subject area	Human molecular genetics
Type of data	Table, gel image, text file
How data was acquired	Applied Biosystems 7300 Real-Time PCR System
Data format	Analyzed data, validated with three independent methods.
Experimental factors	Human blood drop samples and DNA fractions obtained from 45 Japanese individual subjects.
Experimental features	A rapid detection assay of wild-type and whole-gene deletion-type of human *P450 2A6* by multiplex real-time polymerase chain reaction and commercially available TaqMan assays for *P450 2A6* copy number determinations were validated.
Data source location	Showa Pharmaceutical University, Machida, Tokyo, Jc,apan
Data accessibility	The data are supplied with this article.

**Value of the data**

1. A real-time polymerase chain reaction (PCR) assay for *cytochrome P450 2A6* genotyping recently developed [1] was fully validated using a conventional PCR-restriction fragment length polymorphism (RFLP) method (Fig. 1).
2. Aside from the PCR-based methods outlined in this report, commercially available TaqMan assays for copy number determinations (whole-gene deletion P450 2A6*4 genotyping in regions of limited homology) were almost validated (Table 1).
3. Genotyping strategies for *P450 2A6* wild-type (P450 2A6*1) and whole-gene deletion (P450 2A6*4) alleles defined here could be combined with highly automated genome-wide association studies, which have limited utility for identifying whole-gene deletions.
4. The dataset that supports the genotyping validations of human *P450 2A6* wild-type and whole gene deletion are provided by single and multiplex determinations in comparison with commercial TaqMan copy number assays.

## 1. Data

Validation of genotyping of human wild-type P450 2A6*1 and whole-gene deletion P450 2A6*4 by both PCR-RFLP and real-time platforms was carried out. One of the 45 subjects (genotyped as P450 2A6*1B/*1B, Table 1) was not matched in term of *P450 2A6* copy numbers (around 1) in the current TaqMan analysis.

## 2. Experimental design, materials and methods

**Fig. 1:**
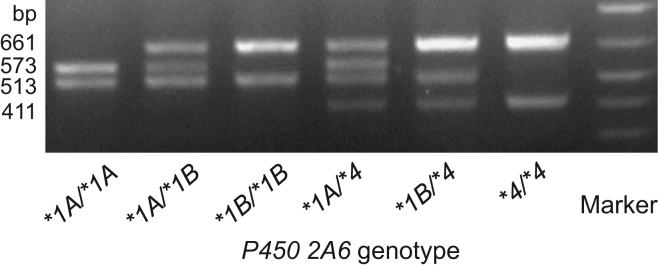
A representative gel image for genotyping of human *P450 2A6* (P450 2A6*1A, 2A6*1B, and 2A6*4) by PCR-RFLP [2], [3], [4] separated on 2% agarose gel. The PCR products of P450 2A6*1A (1323 bp) and 2A6*1B (1322 bp) with a forward primer named 2A6 B4 (5′-CACCGAAGTGTWCCCTATGCTG-3′) and a reverse primer named 2A6 UTR AS-1 (5′-TGTAAAATGGGCATGAACGCCC-3′) [2], [3], [4] were digested by *Fok*I into fragments of 573, 513, 148, and 89 bp and fragments of 661, 513, and 148 bp, respectively. The PCR products of P450 2A6*4 (1309 bp) were digested by *Fok*I into fragments of 661, 411, 148, and 89 bp.

The ethics committee of Showa Pharmaceutical University approved this analysis in accordance with The Code of Ethics of the World Medical Association (Declaration of Helsinki). Informed consent was obtained for experimentation from all subjects. Genotyping of *P450 2A6* (P450 2A6*1A, 2A6*1B, and 2A6*4) was carried out by conventional PCR amplification (Fig. 1) as described previously [2], [3], [4]. Blood samples were obtained with puncture needles, lysed, and stabilized [5]. Blood samples from healthy non-smoking Japanese volunteers or DNA fractions extracted separately by the standard protocol from the volunteers׳ buccal cells were used for real-time detection of *P450 2A6* wild-type and whole-gene deletion [1]. The samples also underwent TaqMan Copy Number Assays (Hs07545274, Hs04488984, and Hs07545275; ThermoFisher Scientific, Waltham, MA, USA). The *P450 2A6* copy numbers in the human genomes were calculated using CopyCaller software according to the manufacturer׳s instructions with designed human *RNase P* TaqMan Copy Number Reference Assays (4403326; ThermoFisher Scientific) after duplex real-time PCR reactions.

## Conflicts of interest

None.

## Figures and Tables

**Table 1:** Comparison of genotyping of *P450 2A6* of 45 individuals in a Japanese population by conventional PCR (Fig. 1), multiplex real-time PCR [1], and three positions for TaqMan methods (07545274, 04488984, and 07545275).

PCR-RFLP, *Fok*I	Multiplex real-time PCR	TaqMan			PCR-RFLP, *Fok*I	Multiplex real-time PCR	TaqMan		
07545274	04488984	07545275	07545274	04488984	07545275
*1A/*1A	*1/*1	*1.8*	*1.9*	*2.4*	*1B/*1B	*1/*1	*1.8*	*1.9*	*2.2*
*1A/*1A	*1/*1	*2.0*	*1.9*	*2.3*	*1B/*1B	*1/*1	*1.7*	*2.1*	*2.4*
*1A/*1A	*1/*1	*1.8*	*2.1*	*2.4*	*1B/*1B	*1/*1	*1.9*	*1.8*	*2.4*
*1A/*1A	*1/*1	*2.0*	*2.0*	*2.0*	*1B/*1B	*1/*1	*2.2*	*2.0*	***2.5***
*1A/*1A	*1/*1	*2.0*	*2.1*	*2.1*	*1B/*1B	*1/*1	*2.1*	*1.7*	*2.1*
*1A/*1A	*1/*1	*2.2*	*2.3*	*2.3*	*1B/*1B	*1/*1	*2.2*	***2.5***	*2.1*
*1A/*1B	*1/*1	*1.8*	*1.6*	*1.7*	*1B/*1B	*1/*1	***2.5***	***2.8***	*2.3*
*1A/*1B	*1/*1	*1.9*	*2.0*	*2.4*	*1B/*1B	*1/*1	*2.1*	*2.1*	*2.2*
*1A/*1B	*1/*1	*2.1*	*2.1*	*2.2*	*1B/*1B	*1/*1	*1.7*	*1.8*	*2.2*
*1A/*1B	*1/*1	*2.2*	*1.8*	*2.4*	*1A/*4	*1/*4	*1.1*	*1.0*	*1.2*
*1A/*1B	*1/*1	*1.9*	*1.9*	*2.0*	*1A/*4	*1/*4	*1.4*	*1.4*	*1.2*
*1A/*1B	*1/*1	*1.9*	*2.0*	*2.4*	*1A/*4	*1/*4	*1.3*	*1.3*	***1.5***
*1A/*1B	*1/*1	*1.8*	*1.9*	*2.2*	*1A/*4	*1/*4	*1.0*	*1.2*	*1.0*
*1A/*1B	*1/*1	*1.9*	*2.3*	*2.4*	*1A/*4	*1/*4	*1.0*	*1.3*	*1.2*
*1A/*1B	*1/*1	*1.5*	*1.9*	***2.5***	*1B/*4	*1/*4	*1.1*	*1.0*	*1.3*
*1A/*1B	*1/*1	*2.1*	*2.0*	***2.5***	*1B/*4	*1/*4	*1.2*	*0.9*	*1.4*
*1A/*1B	*1/*1	*2.1*	***2.5***	*1.7*	*1B/*4	*1/*4	*1.2*	*1.3*	*1.2*
*1A/*1B	*1/*1	*2.0*	*2.3*	***2.5***	*1B/*4	*1/*4	*1.2*	*1.3*	*1.3*
*1A/*1B	*1/*1	*1.9*	*2.3*	***2.5***	*1B/*4	*1/*4	*1.0*	*1.2*	*1.1*
*1A/*1B	*1/*1	*2.0*	*2.0*	*2.0*	*1B/*4	*1/*4	*1.0*	*1.1*	*1.2*
*1A/*1B	*1/*1	*1.7*	*2.2*	*1.7*	*4/*4	*4/*4	*0.1*	*0.1*	*0.1*
*1B/*1B	*1/*1	***0.9***	***1.3***	***1.1***	*4/*4	*4/*4	*0.2*	*0.2*	*0.2*
*1B/*1B	*1/*1	*1.6*	*1.9*	*2.2*					

Bold and italic copy numbers obtained by the TaqMan system were not consistent with the genotyping by conventional PCR and multiplex real-time PCR [1]. A representative PCR-RFLP gel after *Fok*I digestion for P450 2A6*1A, *1B and *4 are shown in Fig. 1.
